# The evidence for interventions in early childhood allergy prevention – towards a living systematic review: protocol

**DOI:** 10.12688/f1000research.51490.2

**Published:** 2021-07-15

**Authors:** Uwe Matterne, Christina Tischer, Jiancong Wang, Helge Knüttel, Jon Genuneit, Michael Perkin, Christian Apfelbacher

**Affiliations:** 1 Institute of Social Medicine and Health Systems Research, Otto von Guericke University Magdeburg, Leipziger Str. 44, Magdeburg, 39120, Germany; 2 Institute for Health Resort Medicine and Health Promotion, State Institute of Health, Bavarian Health and Food Safety Authority, Prinzregentenstraße 6, Bad Kissingen, 97688, Germany; 3 University Library, University of Regensburg, Universitätsstraße 31, Regensburg, 93053, Germany; 4 Pediatric Epidemiology, Department of Pediatrics, Medical Faculty, Leipzig University, Liebigstraße 20a, Leipzig, 04103, Germany; 5 Population Health Research Institute, St George's, University of London, Cranmer Terrace, London, SW17 0RE, UK; 6 Family Medicine and Primary Care, Lee Kong Chian School of Medicine, Nanyang Technological University Singapore, 11 Mandalay Road, 308232, Singapore

**Keywords:** Early childhood allergy prevention, randomised controlled trial, living systematic review

## Abstract

**Background: **Research in early childhood allergy prevention (ECAP) is flourishing and new intervention strategies have proven to be promising. Due to the dynamic nature of ECAP, gaps between what is known and how guidelines inform practice are likely. A living systematic review (LSR) can narrow this gap by incorporating new evidence as it becomes available. No efficacy comparisons across various ECAP interventions for similar outcomes have been carried out. Networks of randomised clinical trials can be evaluated in the context of a network meta-analysis (NMA). We aim to establish a LSR on the efficacy and safety of any intervention investigated in randomised controlled trials (RCT) to prevent the occurrence of allergic sensitisation (AS), symptoms or diagnoses of allergic diseases in infancy and early childhood (0-3 years).

**Methods: **A baseline SR will synthesise the evidence from existing SRs of RCTs as well as RCTs not yet considered in these. After completion of the baseline SR we propose to conduct a LSR. Using this methodology, we aim to undertake constant evidence surveillance, three-monthly search updates, and review updates every three months, should new evidence emerge.

**Conclusions: **The ECAP evidence landscape has undergone dramatic transformations and this process is likely to continue. As a response to this, a LSR offers the potential to allow more timely synthesis of new evidence as it emerges. Long gaps between updates of SRs makes it harder for guidelines and recommendations to be up to date. Users of information, such as parents, may be confused if they encounter new evidence that is not part of a trusted guideline. A LSR approach allows us to continuously search the literature and update the evidence-base of existing ECAP interventions resulting in a decreased timespan from evidence accrual to informing clinical practice.

## Introduction

### Rationale

Allergy in children is common. Frequent food allergies (FA) in children include hen’s egg, cow’s milk and peanut. ^1^ Around 10% of children are affected by FA and the incidence is still rising in developing countries. ^1^ Food allergy impacts quality of life. ^2– 4^ Allergic diseases (eczema, ^5– 7^ asthma and hay fever/allergic rhinitis) ^8, 9^ are also highly prevalent and associated with decreased health-related quality of life (HRQOL). ^10^

**Table 1.** Overview of primary ECAP strategies investigated thus far or in progress in RCTs

Intervention route	Example	Mother		Infant/Child		
		Prenatal	If breastfeeding postnatal	At risk	Not at risk	One or more AS, AD
Oral route	Diet/nutrient supplementation	✓ ^109^	✓ ^40^	✓ ^110^	✓ ^67^	✓ ^111^
	Diet/nutrient avoidance	✓ ^112^	✓ ^112^	✓ ^113^	None found	✓ ^22^
	Early allergenic food introduction	n.a.	n.a.	✓ ^20^	✓ ^108^	✓ ^22^
Skin route	Skincare	n.a.	n.a.	✓ ^39^	✓ ^39^	✓ ^114^
Environmental route	House dust mite avoidance	n.a.	n.a.	✓ ^31^	None found	
Pharmaceutical route	Allergen immuno-therapy (AIT)	n.a.	n.a.	✓ ^115^	None found	✓ ^116^
	BCG vaccination	n.a.	n.a.	✓ ^117^	✓ ^118^	
	Oral H1-antihistamines	n.a.	n.a.	n.a.	n.a.	✓ ^119^

ECAP: early childhood allergy prevention, RCT, randomised controlled trial, AD: allergic disease, AS: allergic sensitisation, superscript numbers indicate an example reference.

**Figure 1.**
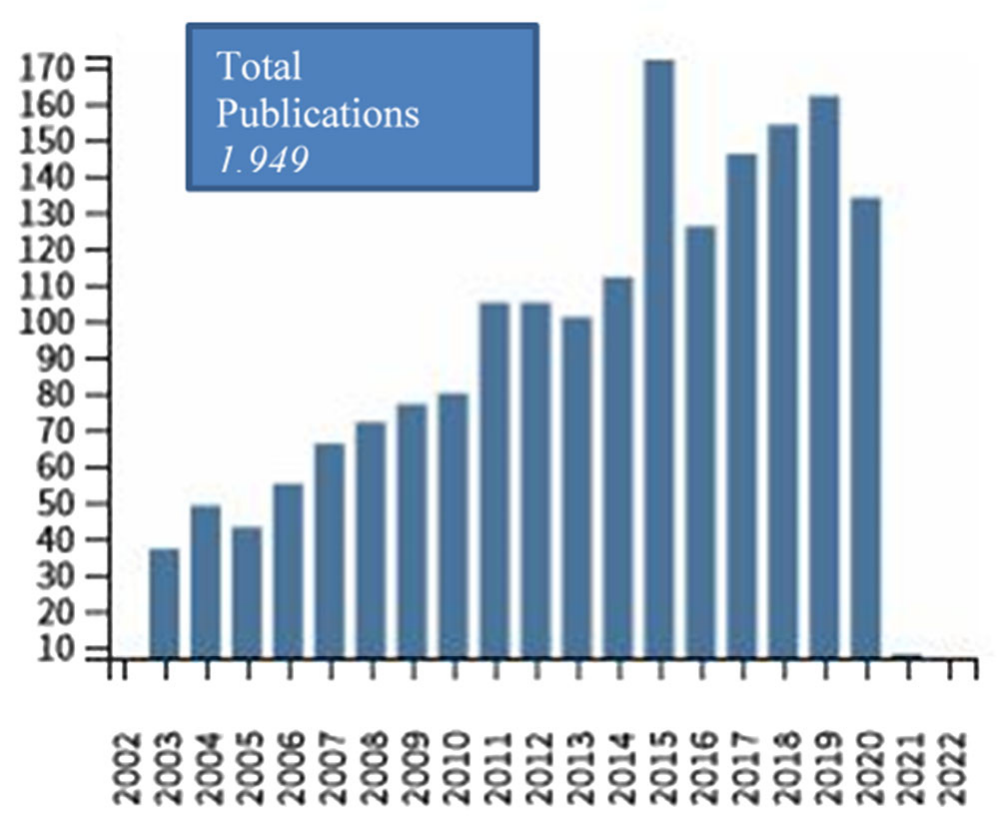
Number of publications derived from Web of Science Core Collection (allergy AND prevention AND childhood) per year since 2002 (performed 26/02/2021).

To counteract the large number of allergies and reduce their burden, a major shift from merely managing manifest allergy to preventing its occurrence has taken place. Previous prevention efforts revolved around avoidance of potential allergens (particularly in at risk individuals), while more recently a new paradigm has been embraced whose focus is on early allergen exposure to induce immune tolerance. This new paradigm has been informing study designs for food allergy prevention. So far it has been established that oral tolerance induction is allergen specific and efficacious in single introduction trials of peanut and egg. ^11^

There is also research activity revolving around environmentally derived prevention paradigms (i.e. exposure to rural environments, cowshed pill, or unpasteurised milk). ^12^ However, one trial found no evidence that an orally applied bacterial lysate affected allergy development in at risk infants. ^13^ It is however, expected that more research in this field will emerge soon.

In general, the design of preventive strategies has been informed by several hypotheses regarding the aetiology of allergy and allergic disease. The original hygiene hypothesis states that lack of exposure to common infections causes allergy. ^14^ However, not all infections protect from allergy, resulting in criticism of the hypothesis. ^15^ Further research has led to modifications of this hypothesis, suggesting that exposure to specific pathogens, commensals and symbionts protects against development of allergy. ^16, 17^ The dual-allergen-exposure hypothesis proposes that exposure to food allergens through the skin leads to allergy, while early consumption of these foods induces tolerance. ^18^ While in the past it was advocated that avoidance of allergenic food would reduce the onset of allergy ^19^ several recent trials have lent support to the dual-allergen-exposure hypothesis. ^20– 22^ The vitamin D hypothesis suggests that low vitamin D levels increase the risk of developing food allergy. ^23, 24^ There are, however, conflicting data on the relationship between vitamin D and the development of food allergy. ^25^

Research in early childhood allergy prevention (ECAP) is flourishing and more than 50 systematic reviews (SR) that exclusively or partly reviewed RCTs ^26– 81^ have been published, some of which provide useful insights. However, not all include a standardised evaluation of the quality of the evidence (e.g. Grading of Recommendations Assessment, Development and Evaluation (GRADE)) ^82^ for randomised controlled trials (RCT). ^83^ Risk of bias (RoB) assessment was done by a variety of different tools. Some used the National Institute for Health and Care Excellence (NICE) methodological checklist ^84^ or the Strength of Recommendation Taxonomy (SORT) ^85^ criteria. Thus, a homogenous RoB assessment and consistent evidence grading approach for each study type would be desirable.

### Research challenges

As intervention effects may vary depending on whether at risk, not at-risk or children with manifest allergy are investigated, studies will be grouped accordingly. ECAP in the latter would still be considered primary prevention, rather than secondary or tertiary prevention, since we would look only at the prevention of new allergies.

Many of the above SRs included observational studies as well as clinical trials. Studies examining the effect of breastfeeding versus no breastfeeding on allergy development cannot use an RCT design; however, for most other ECAP strategies, there are no ethical or other constraints that prevent the use of the gold standard RCT. Hence, we will only consider RCTs in our SR.

Not all ECAP findings may yet be part of allergy prevention guidelines. As outlined above, due to the wealth of studies and new ECAP paradigms, more than 50 SRs have attempted to synthesise the evidence accumulated within the various approaches to ECAP. It is likely that there are gaps between what we know from the best available research and what happens in healthcare practice due to the dynamic nature of ECAP research. The incorporation of new findings into existing reviews or their updates is time-consuming and not always feasible. Hence, adapting prevention guidelines in light of new findings and providing health care providers and other information users with up-to-date evidence may be impeded. Recent advances in the presentation of systematically reviewed (qualitative and meta-analytic) data have led to the concept of a living systematic review (LSR). A LSR is defined as “a systematic review that is continually updated, incorporating relevant new evidence as it becomes available”. ^86^

Furthermore, no efficacy comparisons across various ECAP strategies for similar outcomes have been carried out. Networks of randomised clinical trials can be evaluated in the context of a network meta-analysis (NMA). NMA refers to a procedure that allows inferences about the comparative effectiveness of interventions that may or may not have been evaluated directly against each other. ^87– 89^

### Objective

We aim to establish an LSR on the efficacy and safety of any intervention to prevent the occurrence of allergic sensitisation (AS), symptoms or diagnoses of allergic diseases in infancy and early childhood (0-3 years).

The specific objectives are:

1. To identify all individual-level interventions using the oral, skin, environmental or pharmaceutical route for the prevention of allergy in allergy-free children or the prevention of new allergies in children with manifest allergy
2. To identify all community-level interventions (such as community programmes promoting dietary and environmental diversity in early life) which have thus far been investigated in RCTs to prevent the occurrence of allergy in infancy.
3. To summarise the evidence regarding the effects (efficacy and safety) of these interventions in preventing the occurrence of allergy in infancy and early childhood.
4. To judge the quality of this evidence.
5. To provide a corresponding plain language summary (PLS) accessible for consumers.
6. To develop a workflow for an LSR, which ensures that the evidence synthesis is continuously kept up-to-date.

## Methods

The review will be undertaken according to the methods outlined in the Cochrane Handbook for Systematic Reviews of Interventions, ^90^ PRISMA (Preferred Reporting Items for Systematic Reviews and Meta-Analyses) ^91^ and its extension for application to NMA. ^92^ A protocol registration will be made at PROSPERO. ^93^ Any updates to the protocol will be made through PROSPERO. The protocol was developed closely considering the Prisma-P ^94^ checklist. ^95^ A baseline SR will synthesise the evidence available so far and then transformed into a LSR by regular updates.

### Eligibility criteria

Studies will be eligible for inclusion in the review should they meet the following (PICO) criteria:

- Date of publication: 1980 onward
- Types of study: Randomised controlled trials,
- Population: expectant and/or breastfeeding mothers of and/or children 0 - 3 years (at time of intervention), at risk children (0 - 3 years) (at least one parent with known allergic disease), not at risk children (0-3 years) (no parental allergic disease), children (0-3 years) with manifest allergy (only if intervention aims at preventing a new allergy or study reports this outcome)
- Interventions: any aimed at the individual or community level at preventing the onset of new allergy or allergic disease or allergic sensitisation
  o Oral route (supplements (e.g. vitamins, minerals, pro-, pre-, symbiotic, gut bacteria), time and presence of allergenic food introduction (e.g. peanut, egg protein, cow’s milk, fish), variation in condition (raw, cooked, pasteurised, fermented) and/or amount of complimentary food introduction, avoidance of potential allergens
  o Skin route (e.g. emollients, treated water for washing)
  o Environmental interventions (exposure to natural outdoor environments, green space, outdoor spaces, exposure to farm environments (cow- and other animal-shed bacteria, farming associated bacteria and microbes, bacterial lysate, acinetobacter, microbiome, mucous membrane, microbiota), avoidance of chemicals or allergens (e.g. mattress protector (mites))
  o Pharmaceutical prevention (e.g. allergen-immuno-therapy (AIT), Bacillus Calmette-Guérin (BCG) vaccination))
- Comparator: inactive comparator such as placebo, no intervention or usual care
- Primary outcomes:
  o Physician-diagnosed or parent-reported incidence of allergic asthma (AA), allergic rhinitis (AR), atopic eczema (AE), food allergy (FA)
  o Physician-diagnosed or parent-reported recurrent symptoms of sneeze, wheeze, cough, itch, flexural eczema, or FA
- Secondary outcomes:
  o incidence of AS measured by in-vivo tests such as skin-prick test (SPT)
  o incidence of AS measured by in-vitro tests such as fluorescently labelled anti-IgE antibody, enzyme-linked immunosorbent assay (ELISA), or Radio-Allergo-Sorbent-Test (RAST)
  o adverse events (AE), severe adverse events (SAE), withdrawals

ECAP has revolved around antenatal and postnatal strategies targeting the mother and strategies targeting the child after birth (often during a critical period). Strategies that have been explored in RCTs are illustrated in Table 1.

### Exclusion criteria

Any observational research (cross-sectional, case-control, case-series, prospective/retrospective cohort studies) or quasi-experimental studies (matched controlled designs) will be excluded. We will not consider interventions aimed at treating allergy unless the intervention is also hypothesised to reduce the onset of a new allergic manifestation.

### Search methods

We will use five approaches for the identification of studies for the baseline SR:

1) Topic-based searches in databases and registries
  We will search for all relevant RCTs regardless of publication status (published, unpublished, in press, or ongoing) in the following bibliographic databases: MEDLINE (Ovid), Embase (Ovid), CENTRAL (Cochrane Library), Science Citation Index Expanded & Social Sciences Citation Index (Web of Science), Cochrane Skin Group Specialized Register, GREAT (The Global Resource of EczemA Trials, Centre of Evidence Based Dermatology), in clinical trial registries (U.S. National Institutes of Health ClinicalTrials.gov, ISRCTN registry, Australian New Zealand Clinical Trials Registry, World Health Organization International Clinical Trials Registry Platform, EU Clinical Trials Register).
  We will search the conference proceedings (European Academy of Dermatology and Venereology (EADV), European Academy of Allergy and Clinical Immunology (EAACI), American Academy of Dermatology (AAD), American Academy of Allergy, Asthma & Immunology (AAAI), Asia Pacific Association of Allergy, Asthma and Clinical Immunology (APAAACI) and Asia Pacific Association of Pediatric Allergy, Respirology & Immunology (APAPARI), World Allergy Organisation (WAO)) and Sociedad Latinoamericana de Alergia, Asma e Inmunología, Asunción (SLAAI). Additionally, we will review published documents from health technology assessment agencies (e.g. NICE, IQWIG). No restriction on status or year of publication will be applied. The search will be restricted to publications in English, German, French, Italian or Spanish. An initial draft search strategy for MEDLINE was developed by a medical librarian experienced in comprehensive searches for systematic reviews ( Box 1). The performance of his strategy will be checked against the growing set of known relevant records. The final search strategy will be peer reviewed. The search strategy is composed of the components Population, Intervention, Outcome and Study filter that will be intersected with the Boolean AND operator. For each of the components relevant terms from text fields and controlled vocabulary were used in order to achieve high sensitivity. This strategy will be adapted to the other databases as appropriate.
  Box 1: Initial draft search strategy for MEDLINE
  1 exp infant/ or Child, Preschool/ or (child or children).ti,ab,kf. or (pre-school$ or preschool$).ti,ab,kf. or Nurseries/ or (nursery or nurseries).ti,ab,kf. or exp Parents/ or (parent or parents or mother or mothers).ti,ab,kf. or (infant or infants).ti,ab,kf. or infancy.ti,ab,kf. or toddler?.ti,ab,kf. or (baby or babies).ti,ab,kf. or newborn$.ti,ab,kf. or neonat$.ti,ab,kf. or Pediatrics/ or (pediatric$ or paediatric$).ti,ab,kf. or early childhood.ti,ab,kf. or (Pregnant Women/ or Pregnancy/ or Prenatal Nutritional Physiological Phenomena/) or pregnan$.ti,ab,kf. or Prenatal Exposure Delayed Effects/or Maternal Exposure/or ((maternal or prenatal) adj1 exposure$).ti,ab,kf. or (fetus or fetuses or fetal or foetus or foetuses or foetal).ti,ab,kf. or Fetus/ (Population)
  2 exp Preventive Health Services/ or Preventive Medicine/or "prevention control".fs. or prevent$.ti,ab,kf. or prophyla$.ti,ab,kf. or Infant Formula/or (formula or supplement$).ti,ab,kf. or ((risk or protect$ or development or avoidance or exposure or introduction) adj6 (allerg$ or hypersensitivit$ or atopy or atopic or dermatitis or neurodermatitis or asthma)).ti,ab,kf. (Intervention)
  3 exp Hypersensitivity/ or Allergens/ or allerg$.ti,ab,kf. or hypersensitivit$.ti,ab,kf. or prick test$.ti,ab,kf. or exp asthma/ or Dyspnea/or (asthma$ or dyspnea or wheezing).ti,ab,kf. or (difficult$ adj1 breathing).ti,ab,kf. or rhinoconjunctivitis.ti,ab,kf. or (atopic adj1 (dermatit$ or neurodermatit$ or eczema or disease)).ti,ab,kf. or Diaper Rash/or ((infant or infantile or diaper) adj1 (rash or rashes or eczema or dermatit$)).ti,ab,kf. or Disseminated Neurodermat$.ti,ab,kf. (Outcome)
  4 ((randomized controlled trial or controlled clinical trial).pt. or randomized.ab. or placebo.ab. or drug therapy.fs. or randomly.ab. or trial.ab. or groups.ab.) not (exp animals/not humans.sh.) (Cochrane Highly Sensitive Search Strategy for identifying randomized trials in MEDLINE: sensitivity-maximizing version (2008 revision))
  5 1 and 2 and 3 (Combined concepts: Patients AND Intervention AND Outcome)
  6 5 and 4 (Combined concepts: Patients AND Intervention AND Outcome AND RCTs)
2) Searches by trial registry numbers
  Registry numbers of eligible trials will be collected to be used in follow-up searches with the aim to identify additional trial reports.
3) Trials included in relevant SRs
  We will search for relevant SRs in order to identify additional trials in these SRs.
4) Reference lists of included trials
  We will screen the reference lists of all included study reports. To aid in this process the reference lists will be downloaded from Web of Science when available.
5) Existing systematic reviews
  For the creation of the baseline SR previous SRs on ECAP will be systematically searched for.

### Study selection, data extraction, analyses and syntheses

Database search results will be imported into EppiReviewer (version 4.11.5.3) ^96^ for deduplication and study selection. Assessment of eligibility, data extraction, risk of bias (RoB) evaluation and quality of the evidence assessment will be carried out by at least two researchers independently. The latter will be done according to the GRADE ^82^ recommendations.

The following data will be extracted:

Study characteristics: Author, year of publication, geographical region, study design, type of intervention, type of control, number of participants in intervention group and control group, study duration, time points of assessment, follow-up period, type of primary and secondary outcomes and safety indicators.

Participant characteristics: Person in whom intervention took place (mother (prenatal, postnatal), child, both), age, sex, ethnicity, allergic risk status, parental atopy, presence of allergic sensitisation and/or condition.

Study outcomes: Efficacy outcomes (unadjusted/adjusted): Incidence of allergic sensitization and/or allergic disease; safety outcomes: proportions of AEs, SAEs, withdrawal due to AEs

Existing systematic reviews will be incorporated into the baseline SR. Assessment of the quality of these will be carried out by A MeaSurement Tool to Assess systematic Reviews-2 (AMSTAR-2). ^97^ AMSTAR-2 is a critical appraisal tool for SRs that include randomised or non-randomised studies of healthcare interventions, or both. ^97^ RoB assessment will be done by the use of A Risk of Bias Assessment Tool for Systematic Reviews (ROBIS). ^120^ Findings of SRs whose quality has been judged adequate will be summarised. We intend to publish the results in one or several overviews (umbrella reviews). Conduct of these overviews will follow the methods outlined in chapter 5 of the Cochrane Handbook for Systematic Reviews. ^90^ Only studies of the same intervention and outcome not considered in these SRs and summarised in the overviews will be individually assessed for RoB and quality of the evidence for the baseline SR.

### Meta-analyses and network-meta-analysis (NMA)

If no obvious qualitative heterogeneity within studies exists, we will perform meta-analyses across similar applications of interventions. Pair-wise meta-analyses between two intervention conditions (provided at least two eligible studies exist that are not too heterogeneous) for each different endpoint/outcome (FA, AS, AE, asthma, AR) will be conducted. These are to be updated in the LSR as relevant new studies emerge. To guard against potential type I error inflation and occurrence of type II errors which are a function of the number of analyses done with the same data (as new data is incorporated with each update) we will follow the recommendations outlined by Simmonds *et al*. ^98^

Pairwise meta-analysis is a statistical technique for quantitatively synthesising similar studies in a systematic review. Useful in its own right, it is, however, limited in that it can only compare two interventions simultaneously, and only those evaluated directly in head-to-head trials. ^89^ Pairwise meta-analysis allows for comparisons between pairs of interventions (an experimental intervention and a comparator intervention) for a specific outcome in a particular population or setting. It is, however, often the case that a variety of different interventions are available for any given condition. A single SR that includes all relevant interventions and presents their comparative effectiveness and potential for harm would help people to decide between alternative interventions. NMA affords an analysis option for such a review. ^88^ A network of interventions consists of any set of studies that links three or more interventions via direct comparisons. Within a network, there can be numerous ways to make indirect comparisons between interventions. They refer to comparisons that have not been made directly within studies. Mathematical combinations of the available direct intervention effect sizes are used to estimate indirect effects. In NMA direct and indirect estimates across a network of interventions are combined in a single analysis. ^88^

We also aim to conduct NMA to compare different interventions for the same endpoint/outcome (PO, SO and AE), should assumptions for NMA be met. We will also perform a full evaluation of the confidence in the results from NMA by using the web application CINeMA (Confidence in Network Meta-Analysis). ^99^ This web application simplifies the evaluation of confidence in the findings from NMA and has evolved out of the GRADE approach. The GRADE ^82^ approach offers an assessment of the confidence in the results from systematic reviews and meta-analyses. Many organisations, for example the World Health Organisation, have adopted the GRADE approach. ^100^ Based on GRADE, two systems have been proposed to evaluate the credibility of results from NMAs, ^101, 102^ but the complexity of the methods and lack of suitable software have limited their wide adoption. ^103^ CINeMA is based on six domains: within-study bias (referring to the impact of RoB in the included studies), across-studies bias (publication or reporting bias), indirectness (relevance to the research question and transitivity), imprecision (comparing the range of treatment included in the 95% confidence interval with the range of equivalence), heterogeneity (predictive intervals), and incoherence (if estimates from direct and indirect evidence disagree). ^111^ Judgements across the six domains are then summarised to obtain four levels of confidence for each relative treatment, corresponding to the usual GRADE ^82^ approach: very low, low, moderate or high.

If possible, we will also perform subgroup analysis (sex, atopic predisposition as a marker of high risk to develop allergic sensitization or allergic disease). Prior to this, sources of heterogeneity across studies will be investigated and the impact of inclusion of studies at various risk of bias in meta-analyses will be examined.

### Transformation into a LSR

This review will continuously evaluate the role of interventions for the prevention in early childhood (ECAP). A living systematic review is a cumulative synthesis that is updated regularly as new evidence becomes available. ^86^ According to Cochrane’s Living Evidence Network ^104^ transformation into a LSR is justified when the review question is a particular priority for decision-making, there is an important level of uncertainty in the existing evidence and there is likely to be emerging evidence that will affect the conclusions of the LSR. The review question is of particular priority for decision making because one SR on ECAP ^105^ is among the Cochrane Priority Reviews and the rate of publications on ECAP interventions has substantially increased over the past years ( Figure 1) and is expected to continue so. The level of uncertainty remains an issue. A recent SR on interventions for pregnant or breastfeeding women and/or infants concluded that while dietary avoidance of food allergens, vitamin supplements, fish oil, probiotics, prebiotics, synbiotics, and emollients may have little to no effect on preventing food allergy, the evidence was judged as very uncertain. ^43^ New evidence is expected to emerge on variations of the induction of tolerance paradigm as paved by previous trials such as EAT ^20^ or LEAP. ^22, 106^

An artificial intelligence algorithm for automated searches will be developed in collaboration with information specialists and software engineers. We will gradually incorporate more and more intelligent software tools for eligibility tests, data extraction, and analysis and synthesis presentation as they prove to be reliable in the subsequent update processes.

This evolving search algorithm is to be run at three-monthly intervals and will be set up to notify the author team about studies with a high likelihood of being eligible. At the same interval manual searches will be conducted until the automated search algorithm is sufficiently reliable. The author team will review the search results, decide upon inclusion, and update the living SR’s web version after a new evidence synthesis is deemed necessary every three months. At the same time references of included studies and the corresponding tables and figures will be updated. Every three months the date of each subsequent search, the number of new included studies, and new summary of findings tables will be published on the LSR’s website along with an updated plain language summary.

### Discontinuation of the ‘living’ aspect of the LSR

The LSR will be maintained in its ‘living’ form until no new evidence is likely to arise and/or so long as the certainty of the evidence for particular ECAP interventions remains unsettled. The necessary future funding will be sought.

### Dissemination of findings

The baseline SR will be published in a peer-reviewed journal and indexed publication platform that allows the linkage of review updates through versioning of the review publication. A plain language summary will be provided on a project website. This website is intended to also contain graphical and other information for health care providers and the public.

For the LSR, we will consider resubmission to the journal in which the baseline SR is published should the direction of the effect on the critical outcomes change or a substantial modification to the evidence’s certainty occur.

### Study status

At submission of this protocol the final search strategy is being peer-reviewed.

## Discussion

The prevention of allergy in early childhood is important given the high personal burden and societal costs of allergic diseases. The ECAP evidence landscape has undergone dramatic transformations and this process is likely to continue. As a response to this, a LSR offers the potential to undertake a more timely synthesise of new evidence as it emerges. Long gaps in between updates of SRs may make it harder for guidelines and recommendations to be current and up to date. Users of information, such as parents may be confused if they encounter new evidence that is not part of a trusted guideline. A LSR approach allows us to continuously search the literature and update the evidence-base of existing ECAP interventions resulting in a decreased timespan from evidence accrual to informing clinical practice.

It is also crucially important to assess whether an ECAP intervention is associated with harm. For instance, there is evidence suggesting that early egg white powder introduction at 4 to 6 months of age for the prevention of egg allergy in children from the general population may increase the occurrence of allergy. ^108^

Further, a consistent assessment of the certainty of the evidence in line with the GRADE recommendation will be carried out across all included studies. This will allow a high level of concordance between the certainty of the evidence and future guideline recommendations.

Whilst our approach of including all interventions for the prevention of all allergies in early childhood may seem ambitious, none of the individual systematic reviews being undertaken at present (e.g. ^33, 34^) will be able to conduct a comparison of multiple interventions (network meta-analysis). The latter provides information regarding the relative treatment effect and the ranking order for multiple treatments for a particular outcome irrespective of whether they were conducted in the same or different trials.

### Research, clinical and policy implications

The baseline and subsequent LSR will identify research gaps needing to be addressed by future research. The clinical implications of the LSR will be the provision of up-to-date information that can dynamically inform national, international clinical and public health guidelines and influence the practice of allergists, paediatricians, ENT specialists, other primary care providers and public health authorities in terms of evidence-based ECAP strategies. The provision of a regularly updated plain language summary will benefit parents as they can easily access a widely visible website with information provided in lay terms. The LSR will have include research evidence from across the world and we hope will have a multinational benefit.

## Data availability

### Underlying data

No underlying data are associated with this article.

### Reporting guidelines

Figshare: PRISMA-P checklist for “The evidence for interventions in early childhood allergy prevention – towards a living systematic review: protocol” https://doi.org/10.6084/m9.figshare.14135450.v1. ^95^

Data are available under the terms of the Creative Commons Attribution 4.0 International license (CC-BY 4.0).
