# Supplementary material for: The evidence for interventions in early childhood allergy prevention – towards a living systematic review: protocol
Source: F1000Res. 2021 Jul 15;10:235. Originally published 2021 Mar 24. [Version 2] doi: 10.12688/f1000research.51490.2 (PMC9924298; doi:10.12688/f1000research.51490.2)
Supplement: Peer review history

# Peer review: Reviewer response for version 2

**DOI:** 10.5256/f1000research.58562.r136277

**Published:** 2022-07-29

**Authors:** Gideon Lack

**Affiliations:** 1 Department of Paediatric Allergy, King's College London and Guy's and St. Thomas' NHS Foundation Trust, London, UK

This is an interesting approach.

 I have a few questions:

1. Network meta-analysis (NMA). It is interesting that comparative efficacy of interventions that have not been evaluated directed against each other can be compared. I think more information is required about this approach. Very simply, is it about comparing the efficacy of a prevention against placebo (or non-intervention) in two different trials showing for example that one intervention is twice as effective as another? More information should be provided about this interesting approach. Does it look at population heterogeneity from the two studies being compared, and how are different levels of risk in different populations adjusted for?
2. Timing of an intervention for prevention in early infancy may be critical. Different studies will intervene at different time points, e.g. at birth, 3 months, and 6 months, and these few months can be critical. How will the NMA take these differences into account?
3. Why is the LSR conducted every 3 months? Is this not too frequent? Most professional bodies will update their guidelines every 3-4 years. Is 3 months really necessary? Would once a year not be sufficient? Can the authors provide any evidence or compelling arguments that systematic reviews conducted at 3-monthly intervals are more valuable than those conducted at annual intervals?
4. Why does the objective only study allergies during the first 3 years of life when most respiratory allergies develop at a later time point, and when food pollen syndrome develops at a later time point? Moreover, early interventions could potentially prevent allergies in the first 3 years of life and these preventative effects may disappear at later time points in life. I would strongly argue that any follow-on studies that go on beyond the first 3 years of life are taken into account. Such a long-term approach is well accepted in studies aiming to prevent cardiovascular events and I would recommend longer term follow up beyond the age of 3 where individual RCTs continue to follow the original population.

Is the study design appropriate for the research question?

Yes

Is the rationale for, and objectives of, the study clearly described?

Yes

Are sufficient details of the methods provided to allow replication by others?

Partly

Are the datasets clearly presented in a useable and accessible format?

Not applicable

Reviewer Expertise:

Food allergy

I confirm that I have read this submission and believe that I have an appropriate level of expertise to confirm that it is of an acceptable scientific standard, however I have significant reservations, as outlined above.

# Peer review: Reviewer response for version 1

**DOI:** 10.5256/f1000research.54665.r86682

**Published:** 2021-06-16

**Authors:** Mirjana Turkalj

**Affiliations:** 1 Faculty of Medicine, Srebrnjak Children’s Hospital, J. J. Strossmayer University of Osijek, Zagreb, Croatia

In the study "The evidence for interventions in early childhood allergy prevention - towards a living systematic review: protocol" the authors propose a new, better and more objective intervention concept in early childhood allergy prevention (ECAP). The authors present concrete and objective methodology and metrics. In fact, living systematic review (LSR) requires regular and continuous updates with the implementation of new knowledge in guidelines and clinical practice.

 - However, there are several drawbacks to this concept; confusing and contradictory messages for the clinician in practice, as well as for patients (parents of children), giving guidelines for treatment that may not be applicable to every patient.

 It is therefore important to emphasize that evidence for interventions in early childhood allergy prevention, in fact, only "current guidelines" that can be changed or modified, and that they are not applicable to every patient. It is important for the authors to emphasize that personalized approach is still crucial in prevention strategy.

Is the study design appropriate for the research question?

Yes

Is the rationale for, and objectives of, the study clearly described?

Yes

Are sufficient details of the methods provided to allow replication by others?

Yes

Are the datasets clearly presented in a useable and accessible format?

Yes

Reviewer Expertise:

allergen immunotherapy,  environment and allergy development

I confirm that I have read this submission and believe that I have an appropriate level of expertise to confirm that it is of an acceptable scientific standard.

## References

[1] : Adherence to Mediterranean Diet and Maternal Lifestyle during Pregnancy: Island-Mainland Differentiation in the CRIBS Birth Cohort. *Nutrients* .2020;12(8) : 10.3390/nu12082179 10.3390/nu12082179 32708050PMC7469061

# Peer review: Reviewer response for version 1

**DOI:** 10.5256/f1000research.54665.r84078

**Published:** 2021-06-02

**Authors:** Carlo Caffarelli

**Affiliations:** 1 Department of Medicine and Surgery, University of Parma, Parma, Italy

The present manuscript describes a project on future systematic reviews for interventions in early childhood allergy prevention. This is an interesting issue. However, some important points need to be clarified.

 Introduction:

- The rationale of the study is focused on dual-allergen-exposure hypothesis. It should be underlined that several strategies have been proposed to reduce the burden of allergic diseases as depicted in Table 1. For example, a multifaceted intervention that reduces exposure to allergens has been shown to be effective in preventing allergy onset. On the other hand, the list of “more than 50 systematic reviews” on early childhood allergy prevention is incomplete. For example see Mastrorilli *et al.* (2020 ^1^). 

Study selection:

- It seems that systematic reviews can only be useful for retrieving papers that have not been identified with other means. It is therefore difficult to understand why you should incorporate and assess existing systematic reviews. This point needs to be discussed and it should be providing possible explanations since it is not an objective of the current project.

Meta-analysis:

- It should be clarified whether a meta-analysis will be done when it results that heterogeneous studies with different design and outcome have been included. It seems that sometimes, this frequent limitation is not considered by meta-analysis. So, a statement on this issue is warranted.

Is the study design appropriate for the research question?

Partly

Is the rationale for, and objectives of, the study clearly described?

Partly

Are sufficient details of the methods provided to allow replication by others?

Yes

Are the datasets clearly presented in a useable and accessible format?

Not applicable

Reviewer Expertise:

NA

I confirm that I have read this submission and believe that I have an appropriate level of expertise to confirm that it is of an acceptable scientific standard, however I have significant reservations, as outlined above.

## References

[1] : Primary Prevention of Allergic Diseases: The Role of Early Exposure to Cow's Milk Formula. *Front Pediatr* .2020;8: 10.3389/fped.2020.00420 420 10.3389/fped.2020.00420 32850536PMC7399633

# Response

**Published:** 2021-07-08

**Authors:** Uwe Matterne

**Affiliations:** Institute of Social Medicine and Health Systems Research, Otto von Guericke University, Germany

*We thank the reviewer very much for having taken the time to critically review our manuscript. We have revised the manuscript taking your suggestions into account and hope to have improved the manuscript sufficiently.*

 Introduction:

- The rationale of the study is focused on dual-allergen-exposure hypothesis. It should be underlined that several strategies have been proposed to reduce the burden of allergic diseases as depicted in Table 1. For example, a multifaceted intervention that reduces exposure to allergens has been shown to be effective in preventing allergy onset. On the other hand, the list of “more than 50 systematic reviews” on early childhood allergy prevention is incomplete. For example see Mastrorilli *et al.* (2020 ^1^ ). 

*Thank you for this valuable comment. We agree with the reviewer, that there are several strategies within each route of administration. Table 1 lists the main routes of administration along with one illustrative example. Table 1 is not meant to be fully comprehensive of all strategies but instead meant to provide an indication of the hitherto used routes of intervention with an accompanying example. We would like to highlight, though, that for the purposes of the manuscript we had not intended to do a systematic search of all potentially relevant reviews. Thus, there may still be other reviews having examined randomised controlled trials of ECAP that are not listed in the manuscript. The suggested example by Mastrorilli et al., though a review, is not a systematic review.*

 Study selection:

- It seems that systematic reviews can only be useful for retrieving papers that have not been identified with other means. It is therefore difficult to understand why you should incorporate and assess existing systematic reviews. This point needs to be discussed and it should be providing possible explanations since it is not an objective of the current project.

*We appreciate the reviewer’s comment very much. The respective section was revised to more fully convey the methodological approach to using existing SRs in the production of the baseline review.*

 Meta-analysis:

- It should be clarified whether a meta-analysis will be done when it results that heterogeneous studies with different design and outcome have been included. It seems that sometimes, this frequent limitation is not considered by meta-analysis. So, a statement on this issue is warranted.

*We are grateful for the reviewer’s comment and would like to stress that in the ‘Meta-analyses and network-meta-analysis’ section it is said, that only similar interventions (tested in RCTs only) and the same outcome will be considered in meta-analyses. We added another sentence  at the end of this section to clarify that we intend to examine the sources of heterogeneity and the impact of inclusion of studies at various risk of bias in meta-analyses.*
